# Validation of a De Novo Health Economic Model for Finerenone in Heart Failure with Left Ventricular Ejection Fraction ≥40%

**DOI:** 10.3390/jmahp14010016

**Published:** 2026-03-11

**Authors:** Tobiasz Lemański, Kerstin Folkerts, Phil McEwan, Paul Mernagh, Mateusz Robert Żemojdzin, Michał Pochopień

**Affiliations:** 1 Clever-Access, Wadowicka 8a, 30-001 Krakow, Poland; mateusz.zemojdzin@gmail.com (M.R.Ż.); michal.pochopien@clever-access.com (M.P.); 2 Bayer AG, Aprather Weg 18a, 42113 Wuppertal, Germany; kerstin.folkerts@bayer.com; 3 Health Economics & Outcomes Research Ltd., Unit A, Cardiff Gate Business Park, Copse Walk, Pontprennau, Cardiff CF23 8RB, UK; phil.mcewan@heor.co.uk; 4 Bayer AG, Müllerstraße 178, 13353 Berlin, Germany; paul.mernagh@bayer.com

**Keywords:** model validation, heart failure with preserved ejection fraction, heart failure with moderately reduced ejection fraction, finerenone, health economic analysis

## Abstract

This study aimed to validate the health economic model for finerenone in the treatment of patients with heart failure (HF) and left ventricular ejection fraction (LVEF) ≥40% in the United Kingdom. A Markov model informed by the pivotal FINEARTS-HF trial compared finerenone + standard of care (SoC) to SoC alone. Cross-validation was performed on the results (life years [LYs] and quality adjusted life years [QALYs]) for the SoC arm against three models in HF with LVEF >40%. External validation compared cardiovascular (CV) mortality and the number of total HF events (hospitalisation for heart failure [HFF] and urgent heart failure visit [UHFV]) against FINEARTS-HF. The model estimated similar discounted outcomes to other models in HF (6.47 vs. 6.63–7.91 LYs and 4.78 vs. 4.63–5.27 QALYs). CV deaths (22 vs. 27) and UHFV events (60 vs. 61) avoided with finerenone were similar between the model and FINEARTS-HF. The broad estimated range of avoided HHF events (205–303 vs. 219 in FINEARTS-HF) was largely driven by baseline patient age. This comprehensive validation exercise demonstrated that the finerenone model accurately estimated observed clinical data and was well aligned in its projections with previous models assessing similar populations.

## 1. Introduction

Heart failure (HF) results from structural or functional abnormalities of the heart that cause elevated intracardiac pressures and/or inadequate cardiac output [1]. Rather than a single diagnosis, it is defined as a clinical syndrome characterised by typical symptoms such as breathlessness, ankle swelling, and fatigue, which may be accompanied by signs such as peripheral oedema [1].

Left ventricular ejection fraction (LVEF), i.e., the percentage of blood pumped out of the left ventricle with each heartbeat, is commonly used to determine the severity of HF [1,2]. An LVEF threshold of 40% is frequently employed to classify between heart failure with reduced ejection fraction (HFrEF) and heart failure with mildly reduced or preserved ejection fraction (HFmr/pEF) [3,4]. HFrEF is a HF phenotype characterised by contractile dysfunction and eccentric hypertrophy for which a range of effective therapies have been established [5]. In contrast, HFmr/pEF is a heterogenous condition involving a multitude of contributing causes and risk factors, for which few therapies have demonstrated efficacy and received regulatory approval [6]. Independent of LVEF, the symptom-based New York Heart Association (NYHA) classification is another HF classification commonly used in clinical practice. It ranges from class I (no limitation of physical activity due to HF) to class IV (symptoms of HF present at rest) [7].

Steroidal mineralocorticoid receptor antagonists (MRA) such as spironolactone or eplerenone are among the medication classes recommended by clinical practice guidelines for reducing the risk of morbidity and mortality in patients with HFrEF; however, their utility is less clear in patients with higher LVEF [1,8]. Finerenone is a non-steroidal and selective MRA, which was initially marketed for the treatment of chronic kidney disease in patients with diabetes and has recently been approved by the United States Food and Drug Administration to reduce the risk of cardiovascular death, hospitalisation for heart failure, and urgent heart failure visits in adult patients with HF and LVEF ≥40% [9,10]. With its lower affinity for glucocorticoid, androgen, and progesterone receptors compared to steroidal MRAs, finerenone offers a lower risk of adverse events (AEs) such as gynecomastia and hirsutism [11]. Furthermore, recent real-world evidence from the TriNetX registry in the USA suggest that, compared with spironolactone, finerenone may offer improvements in survival and lower rates of major adverse cardiovascular events in patients with HFpEF [12].

FINEARTS-HF (NCT04435626) was a pivotal, multicentre, randomised, double-blind, placebo-controlled phase III trial that evaluated the efficacy and safety of finerenone in patients with HF (NYHA class II-IV) and LVEF ≥40% in comparison to placebo and in addition to the usual standard of care (SoC) therapy. Treatment with finerenone resulted in a statistically significant and clinically meaningful reduction in the composite primary endpoint of cardiovascular death and total (first and recurrent) HF events, defined as hospitalisations for HF (HHF) or urgent HF visits (UHFV) [13].

Following the positive results from FINEARTS-HF, finerenone will undergo regulatory and reimbursement submissions to enable access to this treatment for patients with HF and LVEF ≥40%. To facilitate health technology assessment (HTA) and other reimbursement submissions, a de novo health economic model, informed by the results of FINEARTS-HF, was developed to estimate the long-term benefits of treatment with finerenone and demonstrate its clinical and economic value relative to SoC.

The objective of the present study is to assess the validity of the finerenone model, and therefore its ability adequately inform decision making in the HTA and reimbursement space.

## 2. Materials and Methods

The validation described in this manuscript followed the good research practice recommendations from the International Society for Pharmacoeconomics and Outcomes Research (ISPOR) and the Society for Medical Decision Making (SMDM) [14]. Validation exercises included checks of face validity (alignment of the model with the current evidence), internal validity (correct implementation of the model), cross-validity against similar models, and external validity against clinical data from the FINEARTS-HF trial.

### 2.1. Model Description

**Figure 1:**
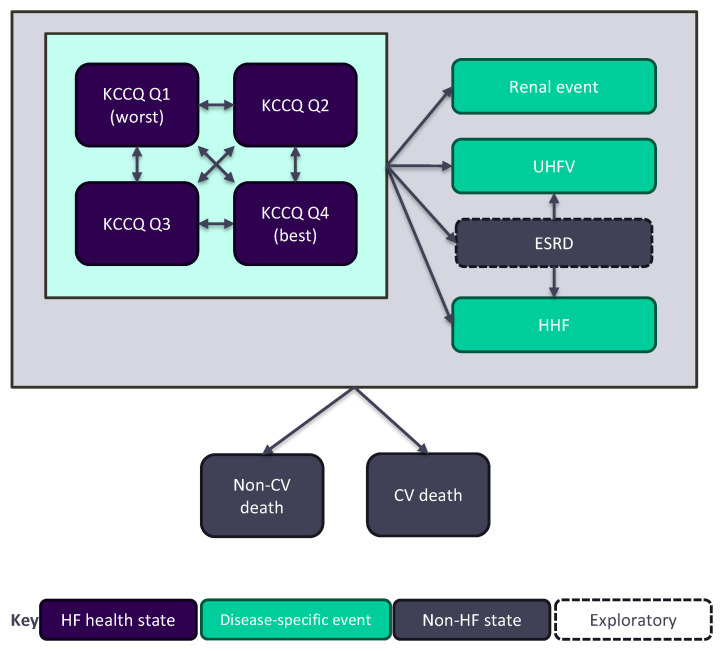
KCCQ-TSS-based structure of the finerenone model. Abbreviations: CV, cardiovascular; ESRD, end-stage renal disease; HF, heart failure; HHF, hospitalisation due to heart failure; UHFV, urgent heart failure visit.

The health economic model for finerenone in HF with LVEF ≥40% is a Markov model with a 4-week cycle length. The model structure and inputs have been reported previously [15]. Briefly, the model features two mutually exclusive alternatives for capturing long-term HF progression and disease severity, with health states based on Kansas City Cardiomyopathy Questionnaire (KCCQ) total summary scores (TSS) (base case, Figure 1) or NYHA class. Ordinal categories based on KCCQ data or NYHA class from the FINEARTS-HF trial were used to inform the starting distribution of patients across health states.

While the NYHA classification is relatively simple and familiar to clinicians, it is subjective and limited to four broad categories that may fail to adequately capture the full spectrum of functional impairment [16,17]. For the purpose of this validation exercise, health states were defined using the KCCQ, in line with recent economic models in HF, including the models for treatments considered in HTAs conducted by the National Institute for Health and Care Excellence (NICE) in the UK [18,19,20,21]. The KCCQ is a patient-reported outcome measure that is commonly collected in clinical trials to assess symptoms, physical and social limitations, and quality of life in patients with HF [22]. The scores range from 0 to 100, with higher scores indicating better health status [22]. To model HF progression, KCCQ TSS scores were divided into quartiles based on the distribution of patients at baseline of FINEARTS-HF (Q1: 1 to <50, Q2: 50 to <69.8, Q3: 69.8 to <87.5, Q4: 87.5 to 100), with each quartile corresponding to a distinct health state (Figure 1). The upper and lower bounds defining KCCQ quartiles were held constant over the model time horizon.

As well as modelling the progression of HF, the model captures the incidence of total (first and recurrent) HF events, including HHF and UHFV. The incidence of HHF and UHFV events is modelled using generalised estimating equations derived from FINEARTS-HF. Further details of the generalised estimating equations are available in the Supplementary Methods.

The safety of modelled therapies is captured through inclusion of AEs with an incidence of ≥1.0% in any treatment group of FINEARTS-HF. This includes hyperkalaemia, an AE of special interest in the context of the mechanism of action of finerenone, as well as pneumonia, atrial fibrillation, COVID-19, COVID-19 pneumonia, acute kidney injury, unstable angina, anaemia, and urinary tract infection. The observed proportion of AEs during the trial were recalculated as a per-cycle probability, based on the median follow-up duration and the 28-day cycle length, assuming that the risk of AEs remains constant over time. This approach provides a pragmatic extrapolation consistent with observed trial data.

Both cardiovascular (CV) and non-cardiovascular (non-CV) mortality are captured in the model by extrapolating the Kaplan–Meier data from FINEARTS-HF using parametric survival modelling. Non-CV mortality is estimated by subtracting the CV death rate from the all-cause mortality rate and is capped by general population estimates of non-CV mortality for the modelled country (for the purpose of this analysis, the UK [23]). If the probability of non-CV death was higher in a given cycle compared to the most recent age- and sex-specific life table probability for the UK general population, the latter was used to inform non-CV death in a given cycle. Life tables were adjusted to exclude CV-related deaths to avoid double counting.

The model assesses finerenone as an add-on to SoC vs. SoC alone, with SoC modelled as a representative basket of treatments commonly used to treat HF with LVEF ≥40%, informed by the baseline distribution of treatments that were administered to patients enrolled in both arms of FINEARTS-HF. The most common medication classes comprising SoC are loop diuretics (87.3%), beta-blockers (84.9%), angiotensin-converting enzyme inhibitors (ACEi) or angiotensin receptor blockers (ARB) (79.3%), and statins (67.5%). Newer therapeutic options that were approved during the course of the trial include sodium–glucose cotransporter-2 inhibitors (SGLT-2i) (13.6%, assumed to be split equally between dapagliflozin and empagliflozin), and angiotensin receptor–neprilysin inhibitors (ARNi) (8.5%). The full composition of SoC is presented in Table S1.

The analyses presented in this manuscript focus on clinical outcomes only. Clinical outcomes estimated by the model include life years (LYs), quality-adjusted life years (QALYs), and the number of total HF events. Although not of interest for this validation exercise, the model also captures cost and cost-effectiveness outcomes, including total costs and disaggregated costs by category, incremental cost-effectiveness ratio (ICER), and net monetary benefit (NMB). Deterministic sensitivity analyses were conducted by varying key model parameters individually across plausible ranges to assess their impact on the results. Probabilistic sensitivity analysis was performed by running the model 1000 times while randomly sampling input parameters from prespecified statistical distributions.

### 2.2. Face and Internal Validity Checks

Assessment of face validity included evaluation of the model type and structure, time horizon and cycle length, and comparator (SoC). Internal validation included a thorough check of all calculations within the model, verification of input values against the sources, and a comparison of model results with a model developed de novo using the same inputs and structure to identify any errors that may have been missed during the calculation check.

### 2.3. Cross-Validation Against Other Models in HF

Cross-validation was performed by comparing the inputs and results for the SoC arm in the finerenone model with three other models: the model for empagliflozin and dapagliflozin developed by Cohen et al. [24], the model for dapagliflozin developed by Booth et al. [25], and the NICE model for dapagliflozin in HFmr/pEF [19]. A comparison between these models is presented in Table 1.

The models captured broadly similar populations (patients with HF and LVEF ≥40% or >40%, i.e., HFmrEF and HFpEF) and compared the intervention of interest added to SoC with SoC alone. However, the exact composition of SoC varied between the studies, including loop diuretics only in the Booth et al. and the NICE models [19,25] and all treatments used in pivotal trials of empagliflozin and dapagliflozin in the Cohen et al. model [24]. It should also be noted that all three models used in the cross-validation process evaluated SGLT-2i in the intervention arm only, while in the finerenone model, SGLT-2i were part of the SoC, and were therefore used in both model arms.

In terms of model settings, the time horizons, cycle length, and baseline population demographics were similar, while discount rates varied, reflecting local guidance or payer preferences. Similar to the finerenone model, Booth et al. and the NICE model for dapagliflozin in HFmr/pEF used KCCQ quartiles to define health states, while Cohen et al. used a distinct model structure. Baseline distribution of patients across health states was only available for the model by Booth et al. and was similar to that in the finerenone model, with <1% differences in occupancy of each health state. In terms of modelling events, mortality and HF events (HHF and UHFV) were modelled using parametric survival curves in the base case finerenone model, the Booth et al. model, and the NICE model for dapagliflozin, while constant rates were calculated in the Cohen et al. model. The approaches to modelling treatment discontinuation also differed, with all three SGLT-2i models using constant rates and the base case finerenone model using parametric curves. The disutilities captured differed slightly between the four models, with the model by Cohen et al. excluding disutilities due to AEs and only capturing disutilities due to HF events after the first year.

To facilitate cross-validation, the finerenone model was adjusted to align with the structure of the Cohen et al. model, allowing for a direct comparison. The primary difference between the two models lay in the mortality assumptions: in the base case, the finerenone model used parametric survival curves, while the Cohen et al. model applied constant probabilities. Therefore, the CV mortality rate of 3.56 per 100 person-years utilised in the Cohen model was converted to a monthly probability of 0.0030 and applied in the finerenone model to adjust it for validation purposes. A similar method was applied for all-cause mortality, which was capped on general population rates. In addition, constant rates of treatment discontinuation and HHF or UHFV probability based on the Cohen et al. model were applied in the finerenone model. Further adjustments included excluding utility decrements for hyperkalaemia and other AEs, as well as omitting disutilities for HF events in the first year. While adjusting the model to the Cohen settings, the baseline utility and disutilities for HHF and UHFV events were adopted from the Cohen et al. model, along with a 3% discount rate.

No adjustments were performed for the purpose of comparisons against the Booth et al. model and the NICE model for dapagliflozin. The finerenone model did not include some of the predictor variables utilised in the Booth et al. model for estimating mortality and probability of HF events (e.g., BMI, presence of type 2 diabetes), making adaptation to the Booth et al. model unfeasible. Adaptation to the NICE model for dapagliflozin was not possible due to limited availability of methodological information, which was largely redacted in the NICE submission.

### 2.4. External Validation Against FINEARTS-HF Results

External validation was performed against on-treatment data from the FINEARTS-HF trial (Bayer AG, Leverkusen, Germany, data on file). Using on-treatment data ensured that patients were analysed according to the intervention they actually received, which may be more relevant for economic evaluations than using an intention-to-treat analysis. Parametric survival modelling based on data from FINEARTS-HF was used to estimate time to all-cause discontinuation of finerenone. Exponential, Weibull, log-normal, log-logistic, Gompertz, and generalised gamma distributions were explored and their fits compared based on the Akaike information criterion (AIC) and Bayesian information criterion (BIC). Treatment discontinuation PSMs were extrapolated over the model time horizon of 28 years (Figure S1). The log-normal curve was selected based on lowest AIC criteria (Table S2). In the model base case, after discontinuation of finerenone, patients were assumed to continue treatment with SoC with the same risks of clinical events and costs as patients in the SoC arm. For the purpose of external validation only, discontinuation rates were set to zero, which was necessary to enable the comparison with the on-treatment dataset from FINEARTS-HF. Without assuming a zero discontinuation rate, the model would have considered patients who switched to SoC following finerenone discontinuation. Setting the finerenone discontinuation rate to zero had no effect on the external validation results for the SoC arm.

The outcomes included in the external validation exercise comprised cardiovascular mortality and the number of total HF events (HHF and UHFV). Both the finerenone + SoC and SoC arms of the model were compared with the results from the corresponding trial arms. HF and UHFV events were modelled based on generalised estimating equations utilising mean age at baseline (a continuous variable, which was varied from 62 to 82 years, i.e., by one standard deviation from the mean age of 72 years in FINEARTS-HF), sex (male, female), and race (White, Asian, Black, Other) as independent variables. For CV deaths, Kaplan–Meier curves from FINEARTS-HF were extrapolated and were not impacted by age or other variables. Half-cycle corrected undiscounted model results (CV death, HHF, and UHFV) were generated for the period of 29 months, corresponding to a median time on treatment of 28 months followed by a 1-month period for reporting events classed as on-treatment in FINEARTS-HF.

In addition to the deterministic estimates, CV mortality, HHF events, and UHFV events were estimated in PSA. Absolute and relative differences between the probabilistic estimates of CV mortality, HHF events, and UHFV events and the rates observed in FINEARTS-HF were calculated.

## 3. Results

### 3.1. Face and Internal Validity

The finerenone model utilises the Markov approach, which has been extensively used in HF modelling in the setting of HTA [18,19,20,21,26,27]. Cycle length was defined as approximately 1 month (28 days), which broadly aligns with the FINEARTS-HF trial data collection schedule for NYHA class and KCCQ (30-day intervals). A monthly cycle is expected to be sufficiently long to capture changes in HF symptoms. The model employs a lifetime time horizon, which is limited to 28 years, as the mean starting age of the population is 72 years. Both parameters align with other models in symptomatic HF assessing for example dapagliflozin [25,28] and empagliflozin/dapagliflozin [24]. The selection of the comparator (SoC alone) was also in line with recent models in HFmrEF and HFpEF considered in HTAs [18,19,27]. Deterministic sensitivity analyses revealed that the most influential parameters on the QALY estimates were transition probabilities between KCCQ-based health states and health state utilities (Figure S2).

A comparison with a model developed de novo using the same inputs and structure resulted in identical results, demonstrating that, following a calculation and input check, the finerenone model did not contain errors which could impact its results.

### 3.2. Cross-Validity

Results for the SoC arm from the finerenone model, the models by Cohen et al. and Booth et al., and the NICE model for dapagliflozin in HFmr/pEF are presented in Table 2.

Patients in the base case finerenone model had longer survival compared to the Cohen et al. and Booth et al. models (12.20 years vs. 7.73 years and 7.8 years, respectively); however, after adjusting mortality inputs, survival projected by the finerenone model (7.77 years) aligned better with the results of Cohen et al. and Booth et al. Undiscounted survival was not reported in the NICE model for dapagliflozin.

The base case finerenone model estimated longer discounted survival than the Cohen et al. model and the NICE model for dapagliflozin (9.39 vs. 6.63 years and 7.91 years, respectively). After adjustment, the finerenone model estimated a mean discounted survival of 6.47 years, which was well aligned with the results of Cohen et al. but lower than the estimate from the NICE model for dapagliflozin. Discounted survival was not reported in the Booth et al. model.

The unadjusted finerenone model estimated a mean of 6.28 discounted QALYs, which was substantially higher than any of the other models (4.63–5.27 QALYs). Following adjustment, discounted QALYs in the finerenone model were well aligned with the estimates of Booth et al. and the NICE model for dapagliflozin (4.78 QALYs vs. 4.63 QALYs and 4.79 QALYs, respectively) but substantially lower than in the Cohen et al. model (5.27 QALYs).

### 3.3. External Validity

Upon external validation of model projections against the results observed in FINEARTS-HF, the model estimates of CV mortality and UHFV were found to align well with trial results (Table 3). For the finerenone + SoC arm, the estimated number of CV deaths (155 in the model vs. 161 in FINEARTS-HF) and UHFV (63 vs. 68, respectively) were similar between the model and the FINEARTS-HF trial. Similarly, acceptable alignment was observed for the SoC arm (CV deaths: 179 vs. 188 and UHFV: 128 vs. 129 for the model and FINEARTS-HF, respectively).

When applying the baseline age from FINEARTS-HF, the model overestimated HHF events (finerenone + SoC arm: 571 vs. 505 HHF events; SoC arm: 842 vs. 724 HHF events for the model and FINEARTS-HF, respectively) (Table 3). Both HHF and UHFV estimates were, however, substantially affected by patient age (Table 3), but not sex or race (Table S3). When patient age in the model was varied from 62 to 82 years (i.e., by one standard deviation according to the baseline distribution of FINEARTS-HF), the resulting ranges of HHF estimates (463–679 for the finerenone + SoC arm and 683–1003 for the SoC arm) included the number of events reported in the trial for both the finerenone + SoC arm and the SoC arm (Table 3). Probabilistic sensitivity analysis resulted in a broad range of HHF events with mean and 95% uncertainty intervals of 532 (473, 593) for finerenone + SoC and 793 (178, 870) for SoC (Table S4). Therefore, the uncertainty intervals include the values observed in the FINEARTS-HF trial.

## 4. Discussion

The finerenone model uses a well-established Markov framework to model the progression of HF and the effect of treatment on disease progression. Health states can be defined based on KCCQ score quartiles or NYHA class, providing flexibility that facilitates model adaptation to different countries and settings. This approach is in line with previous models in HF, which have used both NYHA class [26,29] and KCCQ [18,19,20,21,25,27,30,31] to define HF progression, although the latter was implemented more commonly. The use of KCCQ, a patient-reported outcome measure, to define model health states represents a patient-centred approach, particularly compared with using the NYHA class, which is assigned subjectively by the physician and may not fully capture the functional impairment experienced by the patient [16,17]. However, it should be noted that the KCCQ is focused solely on HF and not on comorbid conditions, which are common in affected patients. As an example, almost 90% of patients in FINEARTS-HF had hypertension, over 40% had type 2 diabetes, while nearly 12% were stroke survivors [13]. Future modelling efforts could explore the use of alternative patient reported outcome measures that account for comorbid conditions, such as the Cardiac and Comorbid Conditions Heart Failure (3C-HF) Score, which has been used to assess the prognosis of patients with HF, as well as activity limitation following discharge from hospital [32,33].

The model was largely informed by FINEARTS-HF, a recent, pivotal, international phase 3 trial providing evidence on the safety and efficacy of finerenone added to usual SoC therapy vs. SoC alone [13]. In FINEARTS-HF, SoC comprised a range of medications used in the treatment of HF, including newer therapeutic options such as ARNi and SGLT-2i that became available only during the course of the trial [13]. The composition of SoC in the model was directly informed by the medications reported at FINEARTS-HF baseline by patients in both trial arms, enhancing the face validity of the model and its relevance to current clinical practice.

Sources of uncertainty were explored using deterministic and probabilistic sensitivity analyses. In DSA, the most impactful parameters on the incremental QALY estimate included transition probabilities and health state utility values. PSA, utilising random sampling of model inputs from appropriate distributions, provided mean estimates of CV mortality, UHFV events, and HHF events that deviated by <20% from the results observed in FINEARTS-HF, suggesting that model results are robust to the uncertainty surrounding input parameters.

External validation of model results performed against the results from FINEARTS-HF demonstrated close alignment in terms of CV mortality and total UHFV events, increasing confidence in the model’s ability to accurately estimate key CV outcomes occurring in patients with HF. However, external validation of HHF estimates proved more complex, as the model overestimated HHF events when mean baseline age from FINEARTS-HF was applied and the results for this outcome were found to be substantially driven by patient age. Unlike FINEARTS-HF, which included patients with a wide range of baseline ages (40–97), a Markov model tracks an entire patient cohort over time without accounting for variability in individual patient characteristics (such as age). Instead, the mean baseline age of the patient cohort is assumed. However, the average predicted number of HHF events across patients of varying ages has proved to differ from the predicted number of events for a patient at the mean age. Moreover, the average age of the modelled cohort increases with every cycle, and with it the risk of HHF, leading to overestimation of the number of events compared with the trial. However, when starting age was varied randomly as part of PSA, the mean probabilistic estimates of the number of HHF events (532 for the finerenone + SoC arm and 793 for the SoC arm) were closer to the number of events observed in the trial (505 and 724 for the two arms, respectively), increasing the confidence in the ability of the model to accurately estimate HHF events. While the consideration about modelling patient age affects both HHF and UHFV estimates in the model, HHF events were far more common than UHFV events, and therefore the absolute difference in the number of events between the model and the trial was more pronounced.

Cross-validation of the results was performed for the SoC arm against three recently published models for SGLT-2i in HFmr/pEF: the NICE model for dapagliflozin and the models developed by Cohen et al. and Booth et al. [19,24,25]. Clinical outcomes (LYs and QALYs) were higher in the base case finerenone model than in any of the other models, which was likely driven by differences in SoC composition, i.e., the inclusion of newer therapeutic options (SGLT-2i, ARNi) in the finerenone model but not the other models. After adjustment of the finerenone model to facilitate a direct comparison with the Cohen et al. model, its results were more similar to those from all three SGLT-2i models. This consistency was observed despite the lack of direct adjustment to the Booth et al. and NICE models and likely arose from all three SGLT-2i models being partly or fully based on the DELIVER trial [34]. Nonetheless, some differences in the projected outcomes were observed. For discounted LYs, the small difference between the Cohen et al. model and the adjusted finerenone model (6.63 vs. 6.47 years, respectively) likely resulted from differences in discounting, which was applied in the first year in the Cohen et al. model but not the finerenone model. Mean discounted QALYs in the Cohen et al. model were substantially higher than in the finerenone model after adjustment. This difference is well explained by the collective impact of structural differences between the models, differences in discounting, fixed utility gains at specific timepoints for patients receiving SoC in the Cohen et al. model, and the much steeper age-related utility decrement in the finerenone model (−0.0051) than in the Cohen et al. model (−0.0007). Specifically, the finerenone model health states are based on the KCCQ, while the Cohen model uses hospitalisation and post-hospitalisation states, resulting in different representations of disease burden and time spent in health states. Furthermore, utilities are applied differently over time: the Cohen model applies an initial utility change during the first year (at 3, 8, and 12 months), with HHF and UHFV disutilities applied only thereafter, while the finerenone model assigns utilities directly based on KCCQ health states. Although less pronounced than for the Cohen et al. model, some differences in discounted QALY estimates were also observed between the adjusted finerenone model and the Booth et al. and NICE models. These could result from slightly different health state definitions (i.e., different cut-offs for KCCQ score quartiles) and health state utilities.

A major strength of this validation exercise is that it followed the rigorous methodology recommended by best practice guidelines from ISPOR and the SMDM [14]. The finerenone model underwent comprehensive internal validation, including input and calculation checks, and performed well in external validation against the FINEARTS-HF trial and in cross-validation against three recent models in HF with LVEF >40%. Overall, the results of validation increase confidence in the projections of the finerenone model and suggest that it accurately reflects the progression of HF and available clinical data on modelled therapies.

Although recommended by best practice guidelines, predictive validity of the finerenone model was not assessed, which is an important limitation of this analysis. Predictive validation involves assessing the ability of the model to predict future outcomes based on a given trial design [14], and was not undertaken due to the limited follow-up duration in FINEARTS-HF (median of 32 months). Indeed, suitable long-term empirical data required for such an evaluation are currently scarce, limiting the feasibility of conducting a robust assessment of predictive validity. Consequently, the validation efforts in this analysis focused on external and cross-validation against other established cost-effectiveness models addressing the same indication, providing a reasonable benchmark in the absence of longitudinal outcome data. Furthermore, additional external validation could potentially be performed in the future against data from the ongoing REDEFINE-HF study (NCT06008197, planned completion in Q2 2026), assessing the efficacy and safety of finerenone compared to placebo in patients who are hospitalised with acute decompensated HF with an LVEF of ≥40%.

## 5. Conclusions

This comprehensive and rigorous validation exercise demonstrated that the finerenone model is correctly implemented, able to accurately estimate observed clinical data, and acceptably well aligned in its projections with previous models assessing similar populations.

The validation results increase confidence in model projections and suggest that it is well suited to serve as a basis for HTA and to inform payer decision making.

## Figures and Tables

**Table 1:** Key features and parameters considered for cross-validation.

Feature/Parameter	Base Case Finerenone Model	NICE Model for Dapagliflozin [19]	Booth et al. 2023 [25]	Cohen et al. 2023 [24]
Model type	Markov	Markov	Markov	Markov
Health states	KCCQ 1 KCCQ 2 KCCQ 3 KCCQ 4 Non-CV death CV death	KCCQ 1 KCCQ 2 KCCQ 3 KCCQ 4 Non-CV death CV death	KCCQ 1 KCCQ 2 KCCQ 3 KCCQ 4 Non-CV death CV death	No HF hospitalisation during simulation, HF hospitalisation, Post-HF hospitalisation, Non-CV death, CV death
Population	Symptomatic heart failure (NYHA class II-IV) with LVEF ≥40%	Symptomatic heart failure with LVEF >40%	Symptomatic heart failure with LVEF >40%	Symptomatic heart failure (NYHA class II-IV) with LVEF >40%
Intervention	Finerenone + SoC	Dapagliflozin + SoC	Dapagliflozin + SoC	SGLT-2i (empagliflozin or dapagliflozin) + SoC
Comparators	SoC	SoC	SoC	SoC
Country	UK	UK	UK ^a^	US
Time horizon	Lifetime	Lifetime	Lifetime	Lifetime
Cycle length	1 month	1 month	1 month	1 month
Perspective	Payer	NHS and PSS	Payer	US health care sector
Annual discount rate (costs and health outcomes) (%)	3.5	3.5	3.5	3
Mean age at baseline (years)	72.0	NR, probably 71.7	71.7	71.7
Proportion of males (%)	54.5	56.1	56.1	55.7
Baseline patient distribution	KCCQ 1: 23.95% KCCQ 2: 26.15% KCCQ 3: 24.55% KCCQ 4: 25:35% Or NYHA I: 0% NYHA II: 69.10% NYHA III: 30.20% NYHA IV: 0.70%	NR	KCCQ 1: 24.7% KCCQ 2: 26.8% KCCQ 3: 24.1% KCCQ 4: 24.4%	NR
Survival	Parametric curves based on the FINEARTS-HF trial	Parametric curves based on the DELIVER trial	Parametric curves based on the DELIVER trial	Constant rate
Treatment discontinuation	Parametric curve or constant rate	Constant rate	Constant rate	Constant rate
HHF and UHFV probability	Parametric curves	Parametric curves	Parametric curves	Constant rate
Utilities	Disutilities due to HF events, composite renal outcomes, hyperkalaemia and other AEs included	Disutilities due to HF events and other AEs included	Disutilities due to HF events and other AEs included	Disutilities due to HF events included only after first year, disutilities due to AEs not included

Abbreviations: AE, adverse event; HF, heart failure; HHF, hospitalisation due to heart failure; KCCQ, Kansas City Cardiomyopathy Questionnaire; LVEF, left ventricular ejection fraction; NHS, National Health Service; NR, not reported; NYHA, New York Heart Association; PSS, Personal Social Services; SGLT-2i, sodium-glucose cotransporter-2 inhibitor; SoC, standard of care; UHFV, urgent heart failure visit. ^a^. Results for Germany and Spain were also presented but not used in the cross-validation process.

**Table 2:** Cross-validation results presenting undiscounted LYs, discounted LY, and discounted QALYs for the SoC alone arms of the finerenone, Cohen et al., Booth et al., and NICE dapagliflozin models.

Outcome	LY (Undiscounted)	LY (Discounted)	QALY (Discounted)
Base case finerenone model	12.20	9.39	6.28
Cohen et al. 2023 [24]	7.73	6.63	5.27
Finerenone model adjusted to Cohen 2023 [24] settings	7.77	6.47	4.78
Booth et al. 2023 [25]	7.8	NR	4.63
NICE model for dapagliflozin [19]	NR	7.91	4.79

Abbreviations: LY, life year; NICE, National Institute for Health and Care Excellence; NR, not reported; QALY, quality-adjusted life year; SoC, standard of care. Note: Neither the Cohen et al. model nor the NICE model for dapagliflozin reported undiscounted QALYs.

**Table 3:** Results of external validation against the results from the FINEARTS-HF trial. Estimates of CV mortality, HHF events, and UHFV events are presented for the finerenone + SoC arm, the SoC arm, and the incremental difference between the two arms, for both the trial and the model. Absolute and relative differences refer to the difference between the model projections and trial data.

	Finerenone + SoC	SoC	Incremental
CV mortality			
FINEARTS-HF	161	188	−27
Model (age 62, 82)	155 (155, 150)	179 (179, 173)	−22 (−22, −23)
Absolute difference	6 (6, 11)	9 (9, 15)	5 (5, 4)
Relative difference (%)	3.7% (3.7%, 6.8%)	4.8% (4.8%, 8.0%)	18.5% (18.5%, 14.8%)
HHF events			
FINEARTS-HF	505	724	−219
Model (age 62, 82)	571 (463, 679)	842 (683, 1003)	−253 (−205, −303)
Absolute difference	66 (42, 174)	118 (41, 279)	34 (14, 84)
Relative difference (%)	13.1% (8.3%, 34.5%)	16.3% (5.7%, 38.5%)	15.5% (6.4%, 38.4%)
UHFV events			
FINEARTS-HFF	68	129	−61
Model (age 62, 82)	63 (32, 122)	128 (64, 246)	−60 (−30, −116)
Absolute difference	5 (36, 54)	1 (65, 117)	1 (31, 55)
Relative difference (%)	7.4% (52.9%, 79.4%)	0.8% (50.4%, 90.7%)	1.6% (50.8%, 90.2%)

Abbreviations: CV, cardiovascular; HHF, hospitalisation due to heart failure; SoC, standard of care; UHFV, urgent heart failure visit.

## Data Availability

Bayer commits to sharing upon request from qualified scientific and medical researchers patient-level clinical trial data, study-level clinical trial data, and protocols. Interested researchers can use www.vivli.org to request access to anonymized patient-level data and supporting documents from clinical studies. Data access will be granted to anonymized patient-level data, protocols and clinical study reports after approval by an independent scientific review panel, with scope and conditions laid out as on Bayer-Vivli.

## Supplementary Materials

The following supporting information can be downloaded at: https://www.mdpi.com/article/10.3390/jmahp14010016/s1, Supplementary Methods. Table S1: Proportion of patients receiving individual components of SoC at baseline, based on the FINEARTS-HF trial. Table S2: AIC/BIC values assessing the fit of parametric distributions to finerenone treatment discontinuation data. Table S3: Results of the scenarios testing upper and lower ranges of variables included in the regression for heart failure events. Table S4: Probabilistic sensitivity analysis results for event outcomes (mean and 95% uncertainty intervals) in comparison with FINEARTS-HF trial results. Figure S1. Parametric survival model extrapolations for time to finerenone treatment discontinuation. Figure S2: Deterministic sensitivity analysis results presenting the impact of 20 most influential parameters on the estimate of incremental QALYs for finerenone + SoC vs. SoC alone.
