# Supplementary material for: Validation of a De Novo Health Economic Model for Finerenone in Heart Failure with Left Ventricular Ejection Fraction ≥40%
Source: J Mark Access Health Policy. 2026 Mar 11;14(1):16. doi: 10.3390/jmahp14010016 (PMC13028132; doi:10.3390/jmahp14010016)
Supplement: Supplementary file 1 [file jmahp-14-00016-s001.zip › jmahp-4082279-supplementary.pdf]

Title: Validation of a de novo health economic model for finerenone in heart failure with left ventricular ejection fraction ≥40%

Authors: Tobiasz Lemański, Kerstin Folkerts, Phil McEwan, Paul Mernagh, Mateusz Żemojdzin, Michał Pochopień

Contents

List of Supplementary Tables 1

List of Supplementary Figures 1

Supplementary Methods 2

Generalised estimating equations.. 2

Supplementary Tables 3

Supplementary Figures 7

List of Supplementary Tables

Table S1: Proportion of patients receiving individual components of SoC at baseline, based on the FINEARTS-HF trial 3

Table S2: AIC/BIC values assessing the fit of parametric distributions to finerenone treatment discontinuation data 4

Table S3: Results of the scenarios testing upper and lower ranges of variables included in the regression for heart failure events[a] 5

Table S4: Probabilistic sensitivity analysis results for event outcomes (mean and 95% uncertainty intervals) in comparison with FINEARTS-HF trial results[a] 6

List of Supplementary Figures

Figure S1. Parametric survival model extrapolations for time to finerenone treatment discontinuation.. 7

Figure S2: Deterministic sensitivity analysis results presenting the impact of 20 most influential parameters on the estimate of incremental QALYs for finerenone + SoC vs SoC alone.............. 8

Supplementary Methods

Generalised estimating equations

First events were modelled using generalized estimating equations (GEE) with a binomial distribution and logit link, while subsequent and recurrent events were analysed using GEE with a Poisson distribution and log link. Analyses were conducted in a cycle-based framework, with cycles defined as 3 months intervals. Health state was included as a time-varying covariate measured at beginning of each cycle. Population-level covariates were obtained at baseline, and prespecified interaction terms (treatment by health state, treatment by subgroup, subgroup by health state) were incorporated into initial models and retained if statistically significant based on a Type III Wald test (p-value<0.05). A compound symmetry working correlation structure was specified, with correlations defined within cycles when applicable to account for within-subject clustering. An unstructured working correlation was initially considered; however, estimation resulted in software warnings indicating unstable or biased correlation estimates and a compound symmetry structure was therefore adopted. Complete case analyses were performed, with no imputation. Robust standard errors were used by default.

Supplementary Tables

Table S1: Proportion of patients receiving individual components of SoC at baseline, based on the FINEARTS-HF trial

Drug class	Proportion of patients in FINEARTS-HF
SGLT-2is	13.6%
ARNIs	8.5%
ACEIs and ARBs	79.3%
Beta-blockers	84.9%
Loop diuretics	87.3%
Thiazide diuretics	13.8%
Digoxin	7.8%
Nitrates*	9.6%
Potassium supplements	11.9%
Potassium lowering agents (including binders)	0.2%
Alpha blocking agents	24.7%
Calcium channel blockers	32.8%
Centrally acting antihypertensives	0.0
Aspirin	32.4%
Statins	67.5%
sMRAs	0.1%
Insulins and analogues	11.5%
Other anti-diabetic drugs	27.8%

Abbreviations: ACEi, angiotensin-converting enzyme inhibitor; ARB, angiotensin receptor blocker; ARNi, angiotensin receptor/neprilysin inhibitor; SoC, standard of care; SGLT2i, sodium-glucose cotransporter 2 inhibitor, sMRA, steroidal mineralocorticoid receptor antagonist.

Table S2: AIC/BIC values assessing the fit of parametric distributions to finerenone treatment discontinuation data

Distribution	AIC	BIC
**Exponential**	8399.22	8419.24
**Weibull**	8328.05	8358.09
**Gompertz**	8315.01	8345.05
**Log-logistic**	8324.17	8354.21
**Log-normal**	**8311.53**	8341.57
**Generalised Gamma**	8334.54	8374.59

Abbreviations: AIC, Akaike information criterion; BIC, Bayesian information criterion

Table S3: Results of the scenarios testing upper and lower ranges of variables included in the regression for heart failure events[a]

Variable		Range tested		SOC		Finerenone + SoC	
				HHF	UHFV	HHF	UHFV
Sex	FEMALE	lower value	0.37	858	129	594	67
		upper value	0.54	826	126	572	65
Race	WHITE	lower value	0.63	909	137	629	71
		upper value	0.94	779	119	539	62
	ASIAN	lower value	0.13	831	127	575	66
		upper value	0.20	853	128	591	67
	BLACK	lower value	0.01	840	127	582	66
		upper value	0.02	843	128	584	66
	OTHER	lower value	0.02	839	127	581	66
		upper value	0.04	845	129	585	67

Abbreviations: HHF, hospitalizations for heart failure; SOC, standard of care; UHFV, urgent HF visits.
a. CV deaths were not impacted by race and sex and are therefore not presented in the table.

Table S4: Probabilistic sensitivity analysis results for event outcomes (mean and 95% uncertainty intervals) in comparison with FINEARTS-HF trial results[a]

	Finerenone + SoC	SoC	Incremental
	CV mortality		
FINEARTS-HF	161	188	-27
PSA	155 (132, 182)	180 (155, 206)	-24 (-23, -24)
Absolute difference	6 (29, 21)	8 (33, 18)	3 (4, 3)
Relative difference (%)	3.7% (18.0%, 13.0%)	4.3% (17.6%, 9.6%)	11.1% (14.8%, 11.1%)
	HHF events		
FINEARTS-HF	505	724	-219
PSA	532 (473, 593)	793 (178, 870)	-261 (-244, -277)
Absolute difference	27 (32, 88)	69 (546, 146)	42 (25, 58)
Relative difference (%)	5.3% (6.3%, 17.4%)	9.5% (75.4%, 20.2%)	19.2% (11.4%, 26.5%)
	UHFV events		
FINEARTS-HF	68	129	-61
PSA	59 (43, 78)	120 (98, 147)	-61 (-55, -69)
Absolute difference	9 (25, 9)	9 (31, 18)	0 (6, 8)
Relative difference (%)	13.2% (36.8%, 13.2%)	7.0% (24.0%, 14.0%)	0.0% (9.8%, 13.1%)

Abbreviations: CV, cardiovascular; HHF, hospitalisation due to heart failure; PSA, probabilistic sensitivity analysis; SoC, standard of care; UHFV, urgent heart failure visit.

a. PSA was performed by running the model 1,000 times while randomly sampling input parameters from prespecified statistical distributions.

Supplementary Figures

Figure S1. Parametric survival model extrapolations for time to finerenone treatment discontinuation

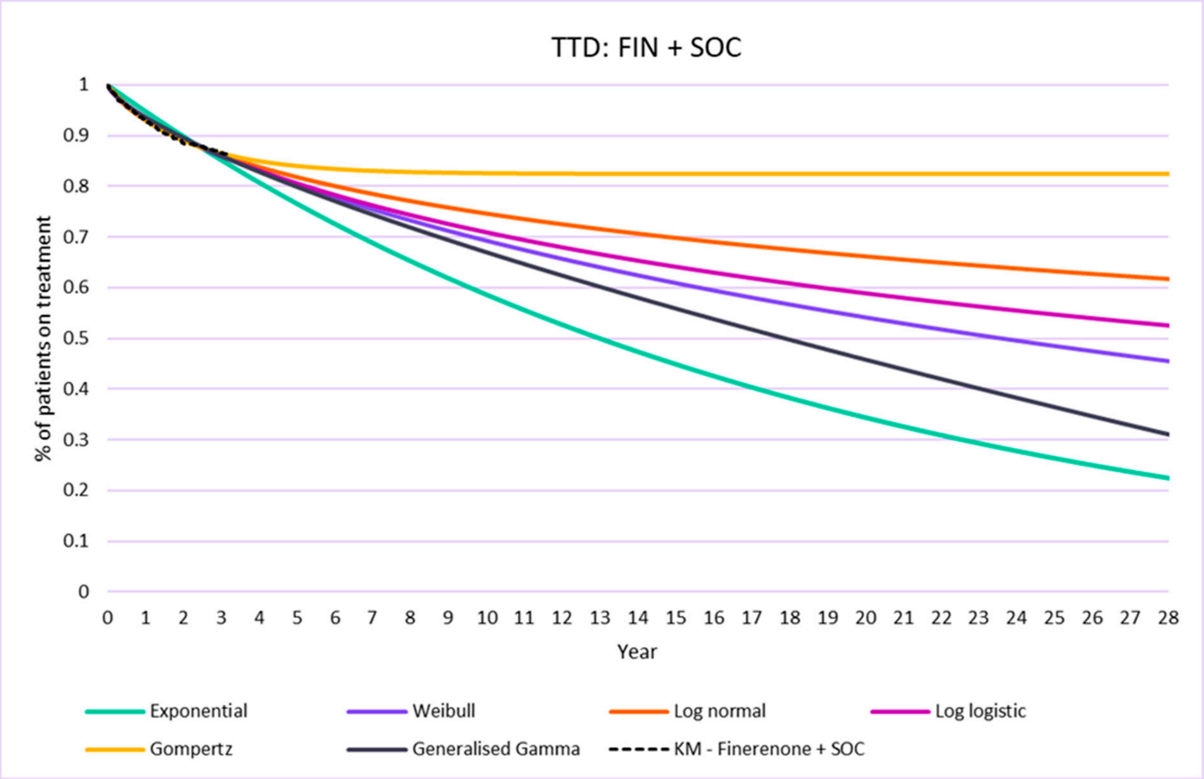

Abbreviations: FIN, finerenone; KM, Kaplan-Meier; SoC, standard of care; TTD, time to discontinuation.

Figure S2: Deterministic sensitivity analysis results presenting the impact of 20 most influential parameters on the estimate of incremental QALYs for finerenone + SoC vs SoC alone

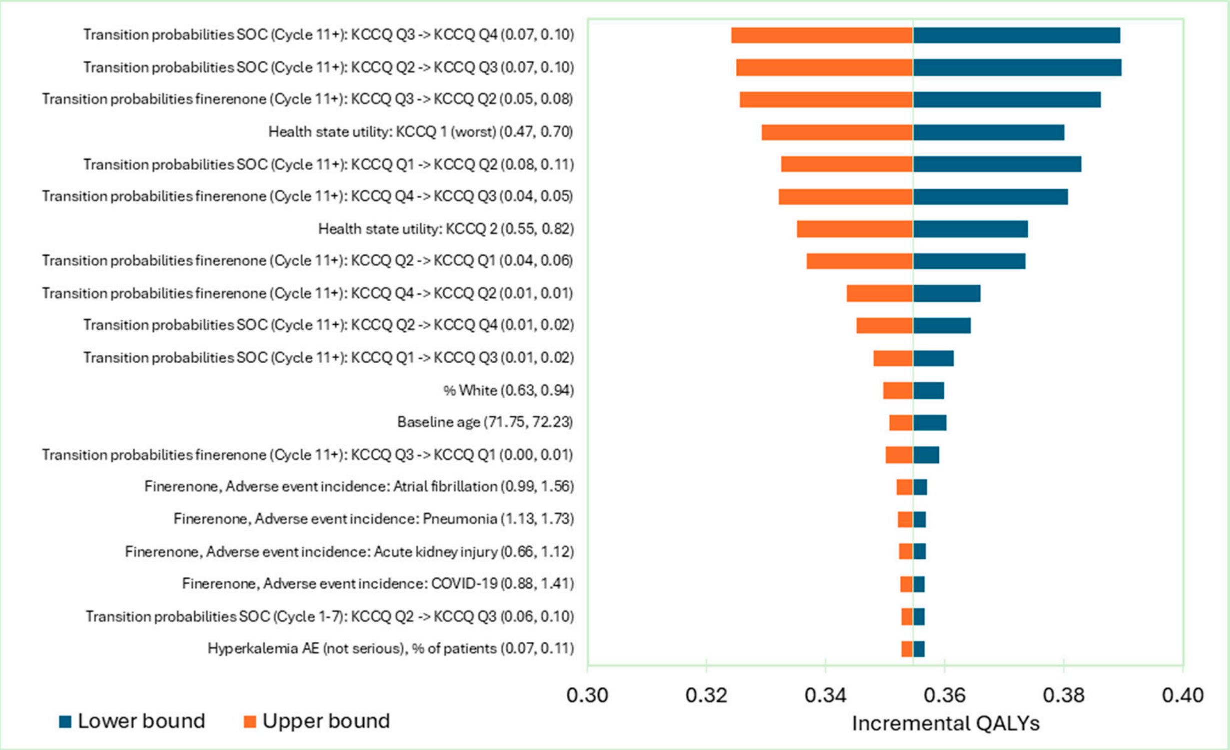

Abbreviations: AE, Adverse event; QALY, quality-adjusted life year; SoC, standard of care.
